# Poly(vinyl chloride)/Nanocarbon Composites for Advanced Potentiometric Membrane Sensor Design

**DOI:** 10.3390/ijms25021124

**Published:** 2024-01-17

**Authors:** Konstantin Yu. Zhizhin, Evgeniy S. Turyshev, Liliya K. Shpigun, Philipp Yu. Gorobtsov, Nikolay P. Simonenko, Tatiana L. Simonenko, Nikolay T. Kuznetsov

**Affiliations:** N. S. Kurnakov Institute of General and Inorganic Chemistry of Russian Academy of Sciences, 119991 Moscow, Russia; zhizhin@igic.ras.ru (K.Y.Z.); phigoros@gmail.com (P.Y.G.); n_simonenko@mail.ru (N.P.S.); egorova.offver@gmail.com (T.L.S.);

**Keywords:** nanocarbon-filled PVC composites, solid-contact potentiometric membrane sensor, electron transfer film, SWCNTs, fullerene-C60

## Abstract

Polymer nanocomposites filled with carbon nanoparticles (CNPs) are a hot topic in materials science. This article discusses the current research on the use of these materials as interfacial electron transfer films for solid contact potentiometric membrane sensors (SC-PMSs). The results of a comparative study of plasticized poly (vinyl chloride) (pPVC) matrices modified with single-walled carbon nanotubes (SWCNTs), fullerenes-C60, and their hybrid ensemble (SWCNTs-C60) are reported. The morphological characteristics and electrical conductivity of the prepared nanostructured composite films are reported. It was found that the specific electrical conductivity of the pPVC/SWCNTs-C60 polymer film was higher than that of pPVC filled with individual nanocomponents. The effectiveness of this composite material as an electron transfer film in a new potentiometric membrane sensor for detecting phenylpyruvic acid (in anionic form) was demonstrated. Screening for this metabolic product of phenylalanine in body fluids is of significant diagnostic interest in phenylketonuria (dementia), viral hepatitis, and alcoholism. The developed sensor showed a stable and fast Nernstian response for phenylpyruvate ions in aqueous solutions over the wide linear concentration range of 5 × 10^−7^–1 × 10^−3^ M, with a detection limit of 10^−7.2^ M.

## 1. Introduction

Carbon nanostructured materials are of great importance in both theoretical and practical applications due to their extraordinary properties, such as their excellent chemical and physical stabilities, high hydrophobicity, and large surface area [1,2,3,4]. Actual fundamental research has demonstrated the successful application of carbon nanoparticles (CNPs) in modern analytical electrochemistry, both individually and as polymer nanocomposites (PNCs) [5,6,7,8,9]. The remarkable charge transfer capability between heterogeneous phases as well as the ease of deposition on various substrates provide an opportunity to utilize this nanomaterial for the development of a new generation of ion-selective electrodes—solid-contact potentiometric membrane sensors (SC-PMSs) [10]. SC-PMSs are asymmetric electrochemical devices in which an ion-sensitive membrane (ISM) is in contact with the electron transfer layer on one side and with the sample solution to be analyzed on the other side. The operating principle of PNC/CNPs as ion-to-electron transducers is attributed to the high capacitance of the double electrical layer formed at the interface between the nanomaterial film and the ISM [11]. The use of this construction allows PNCs to eliminate the functional disadvantages of conventional ion-selective electrodes while significantly enhancing sensor response stability without compromising selectivity [12]. Therefore, the critical need for reliable SC-PMSs with enhanced analytical characteristics requires the production of advanced electrically active polymer materials. As can be seen from Table 1, current research primarily focuses on the utilization of conducting polymer matrices. However, PNCs based on insulating polymer matrices also offer considerable potential for electrochemical applications. The successful use of these materials as electron transducers is highly dependent on the charge transport within the polymer composite phase [13].

Our research focuses on using CNP-filled poly(vinyl chloride)(PVC) nanocomposites (PVC)/CNPs) as interfacial electron transfer layers in SC-PMSs [21,22]. We chose PVC as the host matrix material because of its many valuable properties, such as low cost, good processability, chemical stability, and low flammability [23]. PVC is an electrically insulating polymer (with a film conductivity on the order of 10^−14^ S/m) [24]. However, the combination of PVC with CNPs allows the formation of stable, electrically semiconducting 3D nanostructures. These materials are superior to analogues made from conductive polymer matrices because they eliminate side reactions that can affect the generated potentiometric response. Nevertheless, only limited information is available on this specific topic. Conversely, numerous publications described the preparation and characterization of PVC)/CNP composites [25,26]. Published research has shown that the addition of CNPs to the PVC matrix changes its electrical conductivity, transforming it from an insulator to a semiconductor. The nature and concentration of nanofillers, as well as the bond between the nanofiller molecules and the polymer matrix, strongly influence the electron transport through the system. Most investigations have been devoted to the development of polymer nanocomposites with MWCNTs [27,28,29,30,31,32], Gr [33,34,35], and GrO [36,37]. Until now, only a few papers have discussed composites of PVC/SWCNTs [38,39,40]. Meanwhile, in the last decade, research on the chemistry of single-walled carbon nanotubes (SWCNTs) and their nanocomposites has shown great promise [41,42,43]. SWCNTs possess a high aspect ratio, large surface area, good stability, and unique metallic or semiconducting electrical conductivity. These properties make them excellent candidates for the development of new sensors [21,44,45,46,47]. As for zero-dimensional fullerene-C60, there are two fundamental ways to incorporate fullerenes into the polymer matrix: covalently or through complex bonding. Covalently introducing fullerenes into the polymer matrix causes a restructuring of the matrix. Non-covalent binding of fullerenes to the polymer substance can result in the formation of complexes with modified properties. It was shown that its incorporation into the PVC backbone resulted in functionalized polymers with good electron acceptor properties [48,49]. Unusual properties of fullerenes as π-conjugated systems have been used to fabricate supramolecular hybrids [50].

In general, the electron transfer properties of PNCs as an interphase layer in SC-PMSs depend on various factors, such as their morphology and composition, the structure of the applied nanofillers, and their intra-particle and inter-particle interactions with the base electrode surface. According to the literature, binary (conductor–insulator) composites containing nanoparticles typically have one of two basic microstructures [51]. The first is the matrix structure, where the nanoparticles (granules) are embedded in and coated by the matrix material, and there is practically no particle–particle contact. The second is a percolation structure, which can be thought of as being made up of two types of granules randomly packed together. In this context, the processing conditions of PNCs have a dramatic effect on their microstructure, thereby determining its electrical conductivity [52]. Polymers have been shown to directly disperse untreated CNTs by π-π and CH-π interactions and entanglement [53]. To achieve a high degree of SWCNT dispersion, two approaches are commonly used: opening the end caps of the tubes or attaching functional groups to their side walls. The cutting of SWCNTs has been carried out by the use of concentrated acids, fluorination, or ultrasonication [54].

This study aims to combine the unique electrical properties of SWCNTs and fullerene -C60 with the good stability of plasticized PVC (pPVC) to produce polymer nanocomposites that are suitable for use as ion-to-electron transducers in SC-PMSs. The preparation and performance of PVC/CNP composite films filled with SWCNTs, fullerene-C60, and their hybrid ensemble are described. The SWCNTs-C60 hybrid filler is shown to be superior to PVC-based nanocomposites containing only individual nanofillers. Finally, the potential use of the PVC/SWCNTs-C60 nanocomposite to fabricate a new SC-PMS for the detection of phenylpyruvic acid (2-oxo-3-phenylpropanoic acid, PPA) is demonstrated. This aromatic monocarboxylic acid (in anionic form) is an intermediate in the metabolism of the proteinogenic amino acid phenylalanine in body fluids. PPA (pK_a_ = 4.66) has been identified as a low-molecular-weight biomarker for diagnosing certain human diseases, such as phenylketonuria, alcoholism, and viral hepatitis [55,56].

## 2. Results and Discussion

### 2.1. Performance Evaluation of PVC Nanocomposites with SWCNTs and Fullerene-C60 

To produce PVC/CNT composites, it is essential to attain a stable CNT suspension for an extended period to guarantee a uniform film. Therefore, it is important to take into account the polarity of both the plasticizer and solvent. Several studies have been conducted to improve the dispersion of fillers through polymers by using the so-called solvent blend approach [29]. This technique comprised three steps: dispersing CNPs in a suitable solvent, mixing it with the polymer solution in another solvent (at room temperature or elevated temperature), and recovering the nanocomposite by precipitation or casting a film. Based on literature reports, for more uniform distributing the fillers within the plasticized pPVC matrix, we chose the ultrasonic-accompanied solvent blending technique based on the use of two different organic solvents—chloroform (CHF) and tetrahydrofuran (THF). A series of nanocomposite films containing PVC, o-NPOE as the plasticizer, and CNPs (SWCNTs and fullerene-C60) were prepared and studied. 

**Figure 1:**
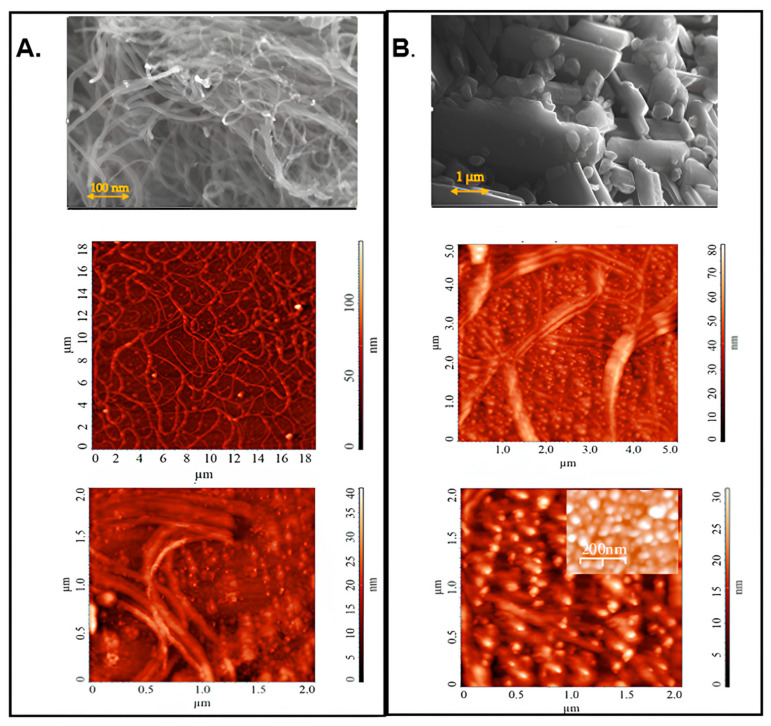
SEM and AFM images obtained for the SWCNTs (**A**) and C_60_ (**B**) used in this work.

*Morphological observations.* The most significant step for the fabrication of conductive PVC-based nanocomposites is the assortment of filler materials. SEM and AFM images obtained for the initial SWCNTs and fullerene-C60 are displayed in Figure 1. 

As can be seen, the morphology of the SWCNT films presents interconnected bundles in highly elongated tubular microstructures with thicknesses ranging from 110 to 300 nm (Figure 1A). The fullerene-C60 film showed two crystalline forms, represented as spherical particles (≤85 nm in size) and flexible microstructures (100–180 nm in thickness) (Figure 1B). 

**Figure 2:**
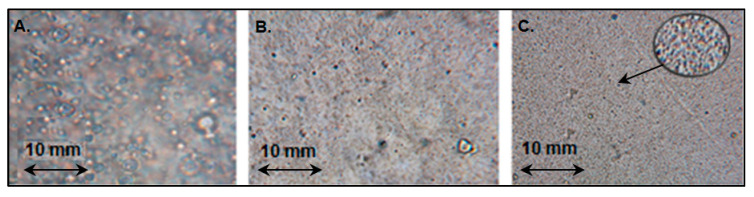
Photographs of the pPVC films produced without sonication and the film loading at 20 °C (**A**) and using ultrasonic irradiation followed by film loading at 20 °C (**B**) and 60 °C (**C**).

Another problem associated with the production of PVC-based nanocomposites is related to the morphology of the virgin PVC matrices. The films formed during PVC precipitation from solutions may have a rough, granular surface. Their morphological traits depend on the production method. Figure 2 displays the effects of ultrasonic irradiation and temperature on the o-NFOE-plasticized PVC film structure. 

It is evident from the relatively smooth surface of the blank pPVC that ultrasonic irradiation had effects on the PVC matrix morphology. Sonication can also affect the capillary absorption process, resulting in the retention of the plasticizer by polymer particles. The optimum conditions for producing a uniform film were reached at 60 °C. 

**Figure 3:**
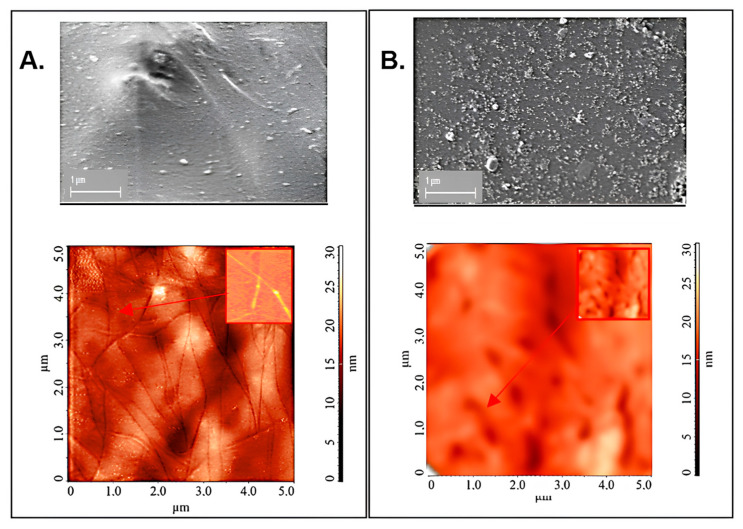
SEM and AFM images obtained for PVC/SWCNT (**A**) and PVC/C_60_ (**B**) composites.

SEM and AFM images of the prepared pPVC/SWCNTs and pPVC/C60 samples are displayed in Figure 3. An observation was made that the nanofillers were dispersed evenly throughout the polymer matrix without any significant agglomeration.

The microstructure of the nanocomposite film that incorporated SWCNTs was characterized by the presence of cavities with a length of at least 3 μm and a width of 50–70 nm which were probably filled with carbon nanotube bundles distributed throughout the material (Figure 3A). The dispersion of SWCNTs can occur through the extraction of individual nanotubes from the bundle or the fracture of individual nanotubes within the bundle. AFM images of the pPVC/C60 film (Figure 3B) mainly show the bulk aggregates (clusters) of fullerene-C60 with a maximum diameter of 200 nm. 

**Figure 4:**
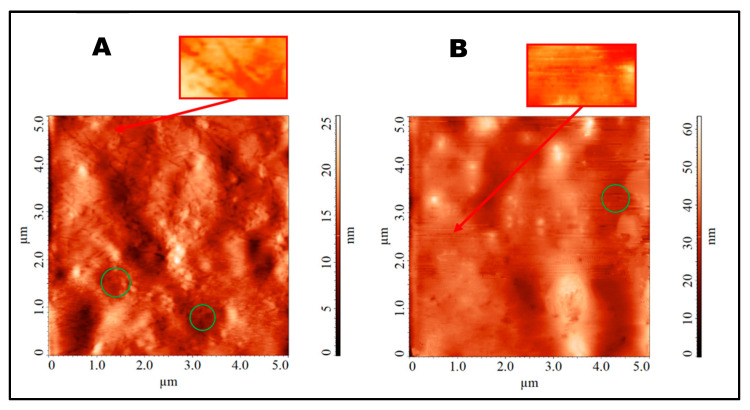
AFM images obtained for the prepared pPVC/CWCNTs-C60 samples, prepared by using a constant amount of C60 (0.3 wt.%) and different amounts of CWCNTs (wt.%): 0.7 (**A**); 1.0 (**B**). The images highlight fragments of the probable interaction of nanoparticles.

As can be seen from Figure 4, the hybrid nanocomposites used, pPVC/CWCNTs-C60, enhanced the dispersion of nanotubes within the pPVC matrix, as evidenced by the reduced number and size of the agglomerates. It can be debated whether the structure of such compositions involves fullerene molecules covering the surface of SWCNTs or being partially encapsulated inside them. The obtained AFM images of the PVC/SWCNTs-C60 samples show that the film structure included tubular motifs covered by formations with diameters of about 120 nm. These bulk agglomerates appear to be C60 agglomerates onto the SWCNT bundles. However, completely homogeneous coverage by C60 was not achieved. 

**Figure 5:**
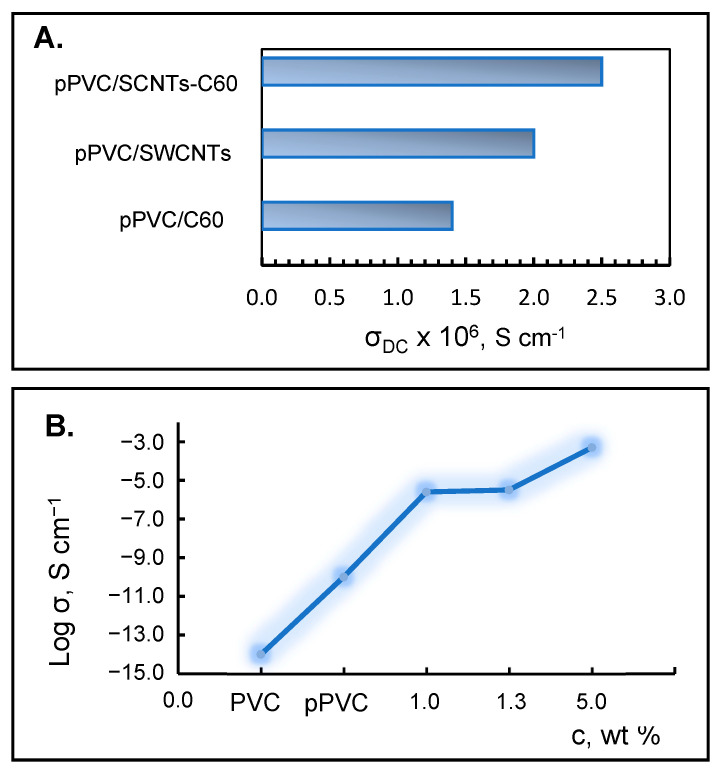
DC conductivity of the prepared PVC-based nanocomposites in the form of thin films. The content of CNPs (wt.%): 1.0 in pPVC/SWCNTs and pPVC/C60; 1.0 (SWCNT: C60 = 7:3) in PVC/SWCNT-C60 (**A**); PNCs with SWCNT: C60 = 7:3 (**B**).

*Electrical conductivity*. Our electrical measurements indicated that modification of a pPVC matrix with SWCNTs and fullerene-C60 increased the conductivity of the nanocomposites prepared in the form of thin films (Figure 5). The specific conductivity of the PVC/SWCNT composite film was higher than that recorded in the case of PVC/C60 (Figure 5A). 

Also, the combination of both types of CNPs in the PVC matrix was found to enhance the film conductivity compared to the individual fillers. This synergistic effect can be attributed to the fact that the strong electronic affinity of fullerene may allow electron transfer from carbon nanotubes to C60 molecules, resulting in a partial positive charge distribution along the nanotube axis. In another words, SWCNTs play the role of molecular wires providing electrical contact. Such a synergistic effect when combining two carbon nanostructures in a polymer composite has already been observed in the literature [32]. 

The concentration dependence of the electrical behavior of PVC/SWCNTs-C60 composite film was investigated (Figure 5B). It was found that the electrical conductivity increased with an increasing number of SWCNTs and amounts of C_60_ in the film. A sharp increase in the DC conductivity of pPVC film by several orders of magnitude (from 1.03 × 10^−9^ S/cm to 5.24 × 10^−4^ S/cm) was observed for a PVC/SWCNTs-C60 (7:3) loading of 5.0 wt.%.

Notably, although SWCNTs have been actively investigated as dopants for PNCs, the DC conductivity of SWCNT-filled PVC composites has been the subject of relatively few studies. It has been suggested that current can flow between carbon nanotubes even when there is no direct contact between carbon particles [39]. The movement of electrons through the insulator between conductive elements can be explained by a mechanism called quantum mechanical tunneling. This mechanism allows electrons to hop from conductor to conductor by ‘tunneling’ through the insulating barrier with a certain probability. The presented results emphasize that PVC/SWNTs-C60 composites are very promising for developing SC-PMSs. SWCNTs-C60 can act as a p-type material. Due to the strong electron affinity of C60, charge can transfer from SWCNTs to C60 molecules, generating a partially positive charge distribution along the axis of the SWCNTs [57,58]. If SWCNTs are heavily filled with C60, a significant charge transfer can occur, greatly increasing the system’s conductivity. When C60 comes into contact with the outer surface of SWCNTs, a comparable charge transfer process may take place, leading to a less significant rise in conductivity.

### 2.2. Application of SWCNTs-C60-Filled pPVC Composite Film for the Development of Potentiometric Membrane Sensor

The integration of low-molecular-weight biomarkers into clinical practice is a current trend in modern medicine. To date, numerous potential markers have been discovered for use in the diagnosis of disease in individuals, but only a few have been applied in clinical practice. The development of portable sensor devices for rapid analysis at the patient’s bedside is of great interest for laboratory diagnostics [59,60]. Early and timely diagnosis is crucial for the survival and recovery of the patient. 

Phenylpyruvate ions (PP^+^), clinically significant metabolites of phenylalanune in the body, are found in large amounts in the biological liquids of patients with phenylketonuria, a serious genetic disorder, and some other diseases. The concentration range of phenylpyruvate in the urine of phenylketonuric patients is 0.3–2 mg L^−1^ (2–12 mM). The determination of PhP^+^ in urine is of significant diagnostic interest in alcoholic disease. 

**Figure 6:**
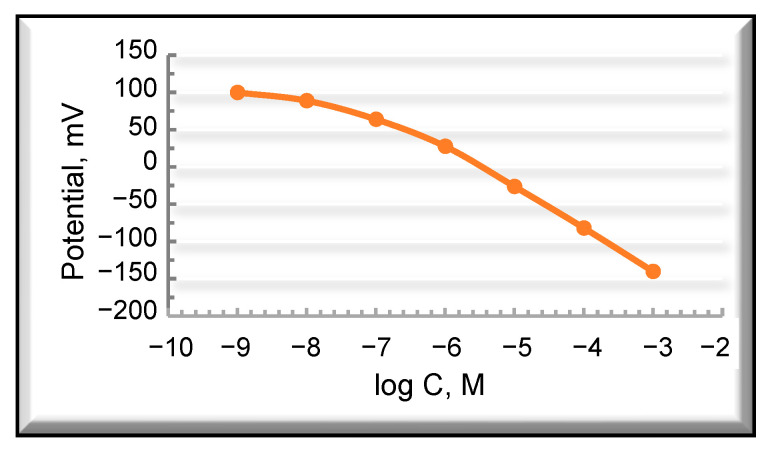
Electrode functions characterizing the potentiometric response of the fabricated SC-PMS towards PPA (PP^+^) concentration in the borate buffer solution (pH = 7.6).

The prepared pPVC/SWCNTs-C60 nanocomposite film was evaluated as the electrically conductive layer in the SC-PMS for detecting PhP^+^ in the borate buffer solution (pH = 7.6). The developed sensor showed a Nernstian potentiometric response with a slope of 57.2 mV/decade in the wide linear range (5 × 10^−7^–1 × 10^−3^ M) with a limit of detection of 10^−7.2^ M (Figure 6). 

The selectivity of the SC-PMS is determined by the ISM composition [61]. The preliminary results show that the new sensor is sufficiently selective against inorganic ions and some other organic metabolites.

The new sensor exhibited a fast response time and stable potentiometric response (Table 2).

In order to determine the analytical characteristics of the new sensor, the standard addition technique was used. The recovery range (n = 5) was found to be 98–102% with a relative standard deviation of less than 3.0%.

It should be noted that only one paper presenting the construction of a phenylpyruvate-selective potentiometric electrode has been published [62]. The primary benefit of the new sensor is the substitution of the internal reference solution with the interfacial electron transport layer. Such a technological design of potentiometric sensors provides lower detection limits, reproducibility, and stability of the electrode response. These features make the sensor more practical for everyday use.

## 3. Methods and Materials

### 3.1. Materials

The commercial carbon nanomaterials, such as fullerene-C60 (≥99.5%, Bucky USA, Baltimore, MD, USA) and SWCNTs (≥90%, Merck, Darmstadt, Germany), were used without any further modification. High-molecular-weight poly(vinyl chloride) (PVC), o-nitrophenyl octyl ether (o-NPOE), chloroform (CHF), tetrahydrofuran (THF), tetradodecylammonium chloride, and phenylpyruvate acid (PPA) were purchased from Sigma-Aldrich (Darmstadt, Germany) or Merck. All chemicals were of analytical or pharmaceutical grade, and their solutions were prepared with redistilled water (resistivity > 18 MΩ·cm^−1^). All solutions were refrigerated between uses.

### 3.2. Equipment

The morphology of the produced PNCs was characterized by light optical microscopy (LOM), atomic force microscopy (AFM), and scanning electron microscopy (SEM). AFM images of the prepared pPVC/CNP composites were recorded using a Solver Pro-M scanning probe microscope (NT-MDT, Zelenograd, Russia) and an NSG-10 silicon probe (NT-MDT, Zelenograd, Russia) (resonant frequency 242 kHz, tip rounding radius < 10 nm) Dualscope DS 95–200, DME atomic force microscope. For static photographs, the digital microscope Bresser LCD (Bresser, Rede, Germany) was used. The surface morphology of CNMs was examined with scanning electron microscope (SEM, Carl Zeiss NVision 40, Oberkochen, Germany). The DC conductivity (σ_DC_) of the polymer films was determined by analyzing the impedance spectra and using the following equation [63]:

σ_DC_ = d/(R_DC_ × S), (1)

where S is the sample area (cm^2^), d is the sample thickness (cm), R_DC_ is bulk resistance (ohm) calculated from the applied voltage. The R_DC_ of the PNC samples was measured by using potentiostat–galvanostat P-45X completed with the impedance-measuring module FRA-24 M operated in the frequency range of 1 MHz–0.1 Hz at 298 K. During the measurements, the samples were fixed between two stainless-steel electrodes.

All potentiometric measurements were conducted using a pH/ion analyzer (Radelkis OP-300, Budapest, Hungary) by using the following galvanic circuit:

CSE|pPVC/CNP film|ISM|Test solution||KCl(satd), AgCl/Ag (2)

The ultrasonic bath (Elmasonic One, Singen am Hohentwiel, Germany, 35-kHz ultrasound) was used in all experiments.

### 3.3. Methods

#### 3.3.1. Preparation of PVC Nanocomposites

The composites of the PVC with SWCNTs and fullerene-C60 were prepared in the form of thin films from the corresponding suspension solutions. The procedure was carried out in four steps, as reported below. In the initial stage, 150 mg of PVC powder was mixed with 350 mg of o-NPOE, and the mixture was dissolved in 7.5 mL of dry freshly distilled THF under effective stirring for 3 h at 60 °C, and then by ultrasonication for 20 min. In parallel, a fixed mass of SWCNTs or C60 (10 mg) was suspended in CHF (2.5 mL) and sonicated for 40 min to reach complete dispersion of the nanoparticles. An SWCNTs-C60 hybrid solution was prepared by dry-mixing the components for 20 min in a mixer heated up to 80–100 °C followed by suspending in CHF. Dispersion of CNPs was characterized using UV-vis absorption spectroscopy at 600 nm, being attributed to the presence of individual CNTs. Then, an appropriate volume of each CNP solution was added to the highly plasticized PVC solution. The resulting mixtures were irradiated in an ultrasonic water bath for 30 min. Finally, each hot mixture was transferred into a glass ring (36 mm in diameter) with a controlled horizontal position. Since the slow evaporation step often leads to re-aggregation of CNP molecules within the composite film, the prepared suspensions were placed on a hot surface under controlled temperature at 60 °C. After controlled solvent evaporation, the resulting films were ca 0.09–0.12 mm in thickness. The films were peeled off from the glass plate and used for further characterization.

#### 3.3.2. Fabrication of SC-PMS

**Figure 7:**
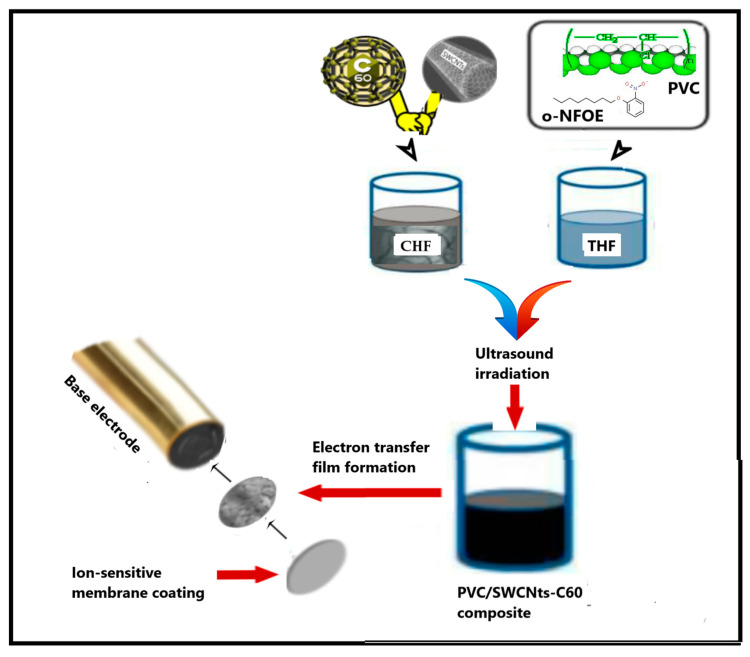
A graphical representation of the SC-PMS preparation protocol.

A potentiometric membrane sensor was fabricated using a carbositall electrode (CSE, Wolta, Russia) as the base conductor (Figure 7). Firstly, an intermediate polymeric layer (electron transfer film) was formed by depositing 10 μL of the PVC/SWCNTs-C60 suspension (in 5 μL aliquots) onto the surface of the CSE. Next, the modified CSE was coated with a membrane cocktail solution (in two portions of 10 μL) to form the ISM film (with a thickness of approximately 150–200 μm). The ion-sensing membrane consisted of the following components (wt.%): PVC—29.0, recognizing lipophilic compound tetradodecylammonium phenylpyruvate)—2.0, and plasticizer (o-NPOE)—69.0. The components were dissolved in 2.0 mL of freshly distilled THF. After the membrane cocktail was homogeneously mixed, it was stored at 4 °C. 

**Figure 8:**
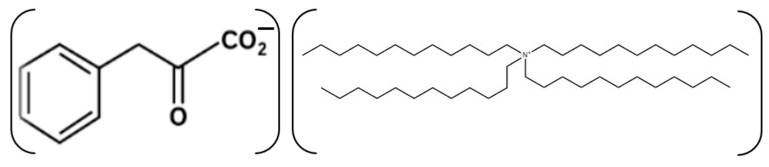
Schematic representation of the complex of tetradodecylammonium phenylpyruvate.

The ion pair association complex of tetradodecylammonium phenylpyruvate (Figure 8) was prepared from tetradodecylammonium chloride using a liquid/liquid ion exchange process.

Before use, each sensor was soaked in 1.0 × 10^−4^ M phenylpyruvate solution (pH 7.6) for 2 h to establish membrane/sample solution equilibrium. The sensors were stored dry in an opaque, closed vessel when not in use.

## 4. Conclusions

In summary, laboratories worldwide have contributed to the development of fundamental and applied aspects of polymer/CNP composites. The field of electrically active polymers for SC-PMSs is far from the maturation phase, which allows finding a solution to many issues, one of which is improving their performance and long-term stability. CNPs are considered the optimal choice for PVC-based polymers due to their exceptional properties. The demand for these materials is increasing by the day. Specifically, the development of advanced electroactive composites incorporating carbon nanomaterials in PVC matrices has become an interesting concept in the field of electrochemical sensors. This study has given some evidence of using CNP-filled PNCs for the development of SC-PMSs with improved analytical characteristics. Today, there is relatively little literature available regarding this topic. Our experiments showed that the use of hybrid nanostructures like SWCNTs- fullerene-C_60_ in a pPVC matrix is possibly the most promising strategy for fabricating such ion sensors. The combination of two types of carbon nanofillers can produce the synergistic effect related to the improvement in electrical conductivity in comparison to individual fillers. Importantly, further development of this strategy may lead to the development of a new generation of high-performance potentiometric devices.

## Figures and Tables

**Table 1:** PNCs as electron-transferring materials in potentiometric membrane sensors.

PNC Components		Sensing Species	Limit of Detection, M	References
Type of CNPs	Polymer Matrix			
MWCNTs	PEDOT	K^+^	1.0 × 10^−6^	[14]
MWCNTs	POT	K^+^	1.6 × 10^−7^	[15]
MWCNTs-COOH	Nafion	Pb^2+^	6.7 × 10^−9^	[16]
Gr-Carbon black	Fluorinated acrylic copolymer	K^+^	7.5 × 10^−7^	[17]
Gr	Polyaniline	Ca^2+^	5.0 × 10^−8^	[18]
GrO	Polypyrrole	Ca^2+^	2.3 × 10^−7^	[19]
MWCNTs	Polyaniline/Nanofibers	Cl^-^	2.7 × 10^−6^	[20]
Fullerene-C60,	Nafion, PVC	Procaine	1.0 × 10^−7^	[21]

Note: MWCNTs—multi-walled carbon nanotubes; Gr—graphene; GrO—graphene oxide; PEDOT—Poly(3,4-ethylenedioxythiophene); POT—Poly(3-octylthiophene-2,5-diyl).

**Table 2:** Response time and stability of potentiometric response of new SC-PMS in a series of samples containing different concentrations of PPA (pH = 7.6).

Concentration of PPA, M	Response Time, s	Potential Drift, mV			
		24 h	7 Days	1 Month	3 Months
1 × 10^−3^	2.2	≈±0.3	±0.7	±3.6	±5.3
1 × 10^−4^	2.4	≈±0.3	±1.0	±4.0	±5.5
1 × 10^−5^	3.6	≈±0.3	±1.2	±4.2	±6.2
1 × 10^−6^	5.1	≈±0.4	±1.4	±4.5	±6.7
1 × 10^−7^	9.0	≈±0.5	±1.5	±5.6	±8.5

## Data Availability

Data are contained within the article.
